# Characteristics, Determinants, and Outcomes of Traumatic Vertebral and Spinal Injuries: A Retrospective Study in the Al-Qassim Region of Saudi Arabia

**DOI:** 10.7759/cureus.32426

**Published:** 2022-12-12

**Authors:** Mahmoud Dibas, Ahmed Ameer, Mohammad J Atiah, Ahmad Ashraf Ibrahim, Oumar Alkhalifa, Mohammad S Hossain, Nazmus Saquib

**Affiliations:** 1 College of Medicine, Sulaiman Al Rajhi University, Bukaryiah, SAU; 2 Department of Neurological Surgery, Buraidah Central Hospital, Buraidah, SAU

**Keywords:** saudi arabia, traumatic spinal injuries, road traffic accidents, hospital stay, american spinal injury association impairment scale

## Abstract

Background

In Saudi Arabia, traumatic vertebral and spinal injuries (TVSIs) are well-recognized injuries with long-term morbidity and mortality. Al-Qassim is among the five regions in the kingdom with the highest number of TVSIs. Little is known about the characteristics of and outcomes for patients with a TVSI in the Al-Qassim region, and we aimed to explore these further.

Methodology

Electronic medical records of patients with a TVSI admitted to Buraidah Central Hospital between January 1, 2017, and December 31, 2019, were examined. Characteristics, outcomes, and length of stay (LOS) in the hospital acute care were reported for the patients, along with their scores (A through E) on the American Spinal Injury Association (ASIA) impairment scale at admission and at discharge.

Results

The sample included 243 patients with a TVSI (median age 35 years). The majority of the participants were Saudi (70%), admitted due to road traffic accidents (67%), and had an ASIA score of E at admission (83%). The median (interquartile range [IQR]) LOS in acute care was 10.0 (4-18) days. Determinants of a prolonged hospital stay included being non-Saudi, having an ASIA score of A through D at admission, and having associated orthopedic injuries. An ASIA score of A through D at admission was the only significant determinant of having an ASIA score of A through D at discharge.

Conclusions

Road traffic accidents accounted for the majority of TVSIs in Al-Qassim. Not having a normal and preserved function at admission (i.e., ASIA score of A through D) was associated with a prolonged hospital stay.

## Introduction

Traumatic vertebral and spinal injuries (TVSIs) often result in life-long disabilities or death [1,2]. TVSIs can take many forms, such as injury to the spinal cord itself or to the nerve roots [3]. They can also affect the bony structures of the spine or the discs and the surrounding ligaments of the spine [3]. The global incidence of TVSI is 10.5 per 100,000 people, with a higher incidence rate reported among low- and middle-income countries compared to higher-income countries [4].

In 2016, the World Bank and the World Health Organization reported a road traffic accident (RTA) mortality rate of 29 per 100,000 people in Saudi Arabia, which notably exceeded the global RTA mortality rate of 18 per 100,000 people [5]. Furthermore, multiple studies in Saudi Arabia have found a mean age of <40 years among people with a TVSI. This creates a huge burden on the country, given that this age group is the most active and economically productive for any country [6-9]. Most Saudi studies have been conducted in Riyadh and have shown that RTAs constitute 60% to 100% of all TVSIs [6-10]. Among these studies, only a few evaluated the average length of hospital stay for patients with a TVSI [11,12].

Al-Qassim is among the five regions in the kingdom with the highest number of RTAs [13]. However, little is known about the characteristics of and outcomes for patients with a TVSI in the Al-Qassim region. To our knowledge, this is the first study to address the topic in this province. Most studies on TVSI have been conducted in Riyadh [6-10]. While Riyadh is a metropolis with a well-built road system, Al-Qassim is an agrarian region with small towns and villages connected by rural roads. In addition, only a few reports have studied the factors associated with prolonged length of stay (LOS) [14-18], and there have been no studies on the factors associated with the American Spinal Cord Injury Association (ASIA) score in TVSI patients. Hence, in this study, we aimed to report the characteristics of and outcomes for patients with a TVSI in the Al-Qassim region. Moreover, we explored the risk factors for a prolonged LOS in the hospital acute care among those with a TVSI and the factors that determined an ASIA score of A through D at discharge.

## Materials and methods

Data source and participant selection

This was a retrospective study of patients with a TVSI who were admitted to and treated in the neurosurgical department of Buraidah Central Hospital (BCH) in the Al-Qassim region of Saudi Arabia between January 1, 2017, and December 31, 2019. There were no data from 2020 onward because the hospital was transformed into a dedicated COVID-19 hospital for that year. All trauma cases arriving at BCH were diverted to other hospitals, so there were no TVSI cases during that period. This study included records of all patients who sustained a TVSI and continued their treatment in BCH, irrespective of their demographics. Patients who were referred to other hospitals or left BCH against medical advice were excluded from the study. Eligible participants were selected, and their data were entered manually into a predesigned survey. This process took two months (March to April 2021).

The study was approved by the Qassim Region Research Ethics Committee (QREC). Participant consent was waived because there was no contact with or assessment of patients; only hospital records without personal identification were assessed.

The extracted data included age, sex, nationality (Saudi or non-Saudi), year of injury, admission and discharge dates from acute care, admission to intensive care unit (ICU; yes or no), mechanism of injury (RTA, fall from height, or other), the ASIA score at admission (A, B, C, D, or E), site of injury (cervical, thoracic, lumbar, and/or sacral), multiple sites (>1), multiple associated injuries (head injuries, chest injuries, orthopedic injuries other than vertebral injuries, visceral injuries, and/or maxillofacial injuries), treatment for spinal injury (conservative, collar or brace, halo vest, or surgery), and the ASIA score at discharge (A, B, C, D, or E). 

The primary outcomes of this study were the ASIA score at discharge and the LOS (defined in days) in acute care. The ASIA score describes the functional ability of a person who has sustained a TVSI and classifies them from A to E: an A score represents complete sensory or motor impairment, and E refers to normal and preserved function [19,20]. Secondary outcomes included (1) functional disability at discharge (none, paraplegia, or tetraplegia), (2) in-hospital mortality, and (3) occurrence of in-hospital complications that involved pulmonary complications (e.g., pneumonia and atelectasis), deep vein thrombosis, bed sores, surgical site infection, or gastrointestinal (GIT) complications.

Statistical analysis

Participants' characteristics and outcomes were expressed using descriptive statistics. Categorical variables were presented as frequencies and percentages and compared using Pearson's Chi-square test or Fisher's exact test. Continuous variables were presented as a median and interquartile range (IQR) and compared using the Mann-Whitney U test. Backward stepwise linear regression and binary logistic regression analyses were conducted to evaluate determinants of a prolonged LOS in the hospital acute care and a discharge ASIA score of A through D (compared to E), respectively. The variables included in the univariable analyses were age, sex, nationality, mechanism of injury, the ASIA score at admission (for the LOS model), admission to ICU, site of injury, and associated injuries. All variables significant to *P* < 0.1 on the respective univariable analyses were included in the final multivariable models. Variables were eliminated from the final models if they were no longer significant to *P* < 0.2. Estimates were presented as beta (β) coefficient for linear regression analyses and odds ratios (ORs) for logistic regression analyses along with their respective 95% confidence intervals (CIs). Linear regression assumptions (including normality, linearity, collinearity, and homoscedasticity) were checked, and age and LOS were log-transformed before including them in the regression models. All analyses were performed using the packages “table1,” “finalfit,” and “dplyr” in R (version 4.0.2, Vienna, Austria). All tests were two-tailed, and *P*-values < 0.05 were considered statistically significant.

Data availability

The datasets generated and/or analyzed during this study are available from the corresponding author upon reasonable request.

## Results

Participants’ characteristics

A total of 243 patients with a TVSI (median age 35 years; IQR 25-46 years; males 80%) were admitted to BCH and included in this study (Table 1). About 37% (91/243) of the patients were admitted in 2019, while 31% (76/243) were admitted in 2017 and 2018. The majority of the participants were Saudi (70%, 169/243), presented due to an RTA (67%, 162/243), and had an ASIA score of E at admission (83%, 194/ 235). Of the total number of participants, 9.5% (23/243) were admitted to the ICU. The lumbar site (56%, 133/237) was the most common injury site, followed by the thoracic (29%, 68/237), cervical (27%, 63/237), and sacral sites (2.1%, 5/237). Orthopedic injuries (19%, 45/243) were the most commonly associated injuries, while visceral injuries (3.3%, 8/243) were the least observed. Of the sample, 44% (92/211) underwent surgical correction of the spinal injury. We compared patients with a TVSI due to an RTA to patients with a TVSI due to a fall from height; the proportion of young Saudi males and the proportion of cervical spine injuries were higher among patients who had had an RTA compared to those who had fallen from height. The proportion of lumbar spine injuries was lower among patients who had had an RTA (Table 2).

**Table 1:** Baseline characteristics of the included participants (N = 243). ^a^Number of available data (i.e., denominator). ^b^Categorical variables are presented as frequencies and percentages, while continuous variables are presented as median and IQR. ASIA, American Spinal Cord Injury Association; ICU, intensive care unit; IQR, interquartile range

	*N*^a^	Values^b^
Age, median (IQR) years	243	34.5 (25-46)
Sex	243	
Male		194 (79.8%)
Female		49 (20.2%)
Year of injury	243	
2017		76 (31.3%)
2018		76 (31.3%)
2019		91 (37.4%)
Nationality	243	
Saudi		169 (69.5%)
Non-Saudi		74 (30.5%)
Mechanism of injury	243	
Road traffic accident		162 (66.7%)
Fall from height		78 (32.1%)
Other		3 (1.23%)
ASIA score at admission	235	
A		8 (3.40%)
B		12 (5.11%)
C		7 (2.98%)
D		14 (5.96%)
E		194 (82.6%)
Admission to ICU	243	23 (9.47%)
Site of injury	237	
Cervical		63 (26.6%)
Thoracic		68 (28.7%)
Lumbar		133 (56.1%)
Sacral		5 (2.11%)
Multiple sites (>1)		32 (13.5%)
Multiple associated injuries	243	74 (30.5%)
Head injuries		19 (7.82%)
Chest injuries		18 (7.41%)
Orthopedic injuries		45 (18.5%)
Visceral injuries		8 (3.29%)
Maxillofacial injuries		9 (3.70%)
Treatment for spinal injury	211	
Conservative		24 (11.4%)
Collar or brace		89 (42.2%)
Halo vest		6 (2.84%)
Surgery		92 (43.6%)

**Table 2:** Comparison between RTAs and falls from height (N = 240). ^a^Number of available data (i.e., denominator). ^b^Categorical variables are presented as frequencies and percentages and were compared by Pearson's chi-square test or Fisher's exact test, as appropriate. Continuous variables are presented as the median and IQR and were compared by the Mann-Whitney U test. ^c^One or more units of an increase/improvement in ASIA score from admission. ^d^One or more units of a decrease/worsening in ASIA score from admission. ASIA, American Spinal Cord Injury Association; ICU, intensive care unit; IQR, interquartile range; LOS, length of stay; RTA, road traffic accident

	*N*^a^	RTAs^b^ (*N *= 162)	Falls from height^b^ (*N* = 78)	*P*-value
Age, median (IQR) years	240	32.0 (23-41)	38.0 (31.3-57)	<0.001
Sex	240			
Male		140 (86.4%)	51 (65.4%)	<0.001
Female		22 (13.6%)	27 (34.6%)	
Year of injury	240			
2017		61 (37.7%)	14 (17.9%)	0.008
2018		45 (27.8%)	30 (38.5%)	
2019		56 (34.6%)	34 (43.6%)	
Nationality	240			
Saudi		130 (80.2%)	38 (48.7%)	<0.001
Non-Saudi		32 (19.8%)	40 (51.3%)	
ASIA scale at admission	232			
A		7 (4.5%)	1 (1.3%)	0.304
B		9 (5.7%)	2 (2.7%)	
C		3 (1.9%)	4 (5.3%)	
D		8 (5.1%)	6 (8.0%)	
E		130 (82.8%)	62 (82.7%)	
Admission to ICU	240	19 (11.7%)	4 (5.1%)	0.158
Site of injury	234			
Cervical		57 (36.3%)	6 (7.8%)	<0.001
Thoracic		45 (28.7%)	23 (29.9%)	0.848
Lumbar		76 (48.4%)	54 (70.1%)	0.002
Sacral		3 (1.9%)	2 (2.6%)	0.665
Multiple sites (>1)		24 (15.3%)	8 (10.4%)	0.306
Multiple associated injuries	240	51 (31.5%)	23 (29.5%)	0.754
Head injuries		15 (9.3%)	4 (5.1%)	0.318
Chest injuries		15 (9.3%)	3 (3.9%)	0.191
Orthopedic injuries		28 (17.3%)	17 (21.8%)	0.402
Visceral injuries		8 (4.9%)	0 (0%)	0.056
Maxillofacial injuries		9 (5.6%)	0 (0%)	0.033
Treatment for spinal injury	209			
Conservative		14 (10.2%)	10 (13.9%)	0.384
Collar or brace		63 (46.0%)	26 (36.1%)	
Halo vest		5 (3.7%)	1 (1.4%)	
Surgery		55 (40.1%)	35 (48.6%)	
ASIA score at discharge	227			
A		4 (2.6%)	2 (2.7%)	>0.999
B		2 (1.3%)	0 (0%)	
C		4 (2.6%)	2 (2.7%)	
D		6 (4.0%)	3 (4.0%)	
E		136 (89.5%)	68 (90.7%)	
ASIA score at follow-up	229			
Improved^c^		13 (8.4%)	8 (10.7%)	0.753
No change		137 (89.0%)	66 (88.0%)	
Worsened^d^		4 (2.6%)	1 (1.3%)	
Functional disability at discharge	240			
None		150 (94.9%)	74 (94.9%)	>0.999
Paraplegia		3 (1.9%)	2 (2.6%)	
Tetraplegia		4 (3.2%)	2 (2.6%)	
LOS, median (IQR) days	240	8.5 (4-17.8)	12.0 (5-19.8)	0.259
In-hospital mortality	240	5 (3.1%)	1 (1.3%)	0.667

Clinical and functional outcomes

Most of the patients (90%, 206/230; Table 3) had an ASIA score of E at discharge. Improvement in the ASIA score at discharge compared to the score at admission was observed among 9.1% (21/232) of the patients; most of the remaining patients had no change in their scores (89%, 206/232). The median (IQR) LOS in the hospital acute care was 10.0 (4-18) days. Complications documented during admission are given in Table 3. In-hospital mortality was 2.5% (6/243).

**Table 3:** Clinical and functional outcomes in traumatic vertebral and spinal injuries (N = 243). ^a^Number of available data (i.e., denominator). ^b^Categorical variables are presented as frequencies and percentages, while continuous variables are presented as median and IQR. ^c^ One or more units of an increase/improvement in ASIA score from admission. ^d^One or more units of a decrease/worsening in ASIA score from admission. ASIA, American Spinal Cord Injury Association; GIT, gastrointestinal; IQR, interquartile range; LOS, length of stay

	N^a^	Values^b^
Complications during admission	243	
Deep vein thrombosis		1 (0.4%)
Bed sores		1 (0.4%)
Surgical site infection		2 (0.8%)
Pulmonary complications (e.g., pneumonia and atelectasis)		6 (2.5%)
GIT complications		2 (0.8%)
ASIA score at discharge	230	
A		6 (2.6%)
B		3 (1.3%)
C		6 (2.6%)
D		9 (3.9%)
E		206 (89.6%)
ASIA score at follow-up	232	
Improved^c^		21 (9.1%)
No change		206 (88.8%)
Worsened^d^		5 (2.2%)
Functional disability at discharge	240	
None		226 (94.2%)
Paraplegia		9 (3.8%)
Tetraplegia		6 (2.5%)
LOS, median (IQR) days	243	10.0 (4-18)
In-hospital mortality	243	6 (2.5%)

Determinants of LOS in acute care and discharge with an ASIA A-D score

The univariable analysis showed that nationality, ASIA score at admission, admission to ICU, and related chest and orthopedic injuries were associated with log-transformed LOS in acute care (Table 4). In the multivariable analysis, being non-Saudi (β 0.65, 95% CI 0.39-0.92, *P *< 0.001), having an ASIA score of A through D at admission (β 0.95, 95% CI 0.63-1.27, *P *< 0.001), and having related orthopedic injuries (β 0.57, 95% CI 0.25-0.89, *P *< 0.001) were the significant determinants of a prolonged LOS in acute care (Table 4).

**Table 4:** Determinants of LOS in the hospital acute care for patients with traumatic spinal injuries. ^a^Age and LOS in the hospital acute care were log-transformed to improve the normality of data and the model assumptions. ^b^Variables were entered into the multivariable linear regression model if they were significant to *P *< 0.1 in univariable regression. Variables were retained if they remained significant to *P* < 0.2. Number of patients in the final model was 235. ^c^The reference group is “no.” #Dropped from the model due to nonsignificance. ASIA, American Spinal Cord Injury Association; ICU, intensive care unit; LOS, length of stay; SD, standard deviation.

	Log-transformed LOS (number of days)^a^					
	Univariable			Multivariable (*R*^2 ^= 0.271)^b^		
	Beta	95% CI	*P*-value	Beta	95% CI	*P*-value
Per SD change in log-transformed age^a^	0.04	–0.28 to 0.35	0.821			
Sex						
Male	Reference					
Female	–0.10	–0.45 to 0.24	0.558			
Nationality						
Saudi	Reference					
Non-Saudi	0.68	0.40-0.96	<0.001	0.65	0.39-0.92	<0.001
Mechanism of injury						
Road traffic accident	Reference					
Falls from height	0.14	–0.15 to 0.44	0.341			
Other	0.57	–0.68 to 1.82	0.372			
ASIA scale at admission						
E	Reference					
A-D	1.06	0.72-1.40	<0.001	0.95	0.63-1.27	<0.001
Admission to ICU^c^	0.67	0.21-1.14	0.005	^#^		
Site of injury						
Cervical^c^	0.12	–0.19 to 0.43	0.450			
Thoracic^c^	0.17	–0.14 to 0.47	0.277			
Lumbar^c^	–0.01	–0.29 to 0.27	0.960			
Multiple associated injuries						
Head injury^c^	–0.01	–0.52 to 0.50	0.971			
Chest injury^c^	0.49	–0.03 to 1.02	0.065		^#^	
Orthopedic injuries^c^	0.80	0.46-1.14	<0.001	0.57	0.25-0.89	<0.001
Visceral injury^c^	0.53	–0.24 to 1.30	0.175			
Maxillofacial injuries^c^	0.46	–0.27 to 1.19	0.215			

The results of the univariable analysis for the determinants of a discharge ASIA score of A through D are shown in Table 5. The multivariable analysis showed that having an ASIA score of A through D (as compared to E) at admission (OR 311.42, 95% CI 57.84-5835.85, *P *< 0.001) was significantly associated with an ASIA score of A through D at discharge (Table 5).

**Table 5:** Determinants of an ASIA score of A-D (as compared to E) at discharge in traumatic spinal injuries. ^a^Variables were entered into the multivariable binary logistic regression model if they were significant to *P* < 0.1 in univariable regression. Variables were retained if they remained significant to *P* < 0.2. ^b^Age was log-transformed before the inclusion of the model to improve the normality of data. ^c^The reference group is “no.” #Dropped from the model due to nonsignificance. ASIA, American Spinal Cord Injury Association; ICU, intensive care unit; SD, standard deviation.

	Discharge ASIA scale of A-D	
	Univariable	Multivariable^a^
	Odds ratio (95% CI, *P*-value)	
Per SD change in log-transformed age^b^	1.27 (0.48-3.38, *P* = 0.632)	
Sex		
Male	Reference	
Female	0.74 (0.21-2.07, *P* = 0.594)	
Nationality		
Saudi	Reference	
Non-Saudi	1.70 (0.70-4.01, *P* = 0.230)	
Mechanism of injury		
Road traffic accident	Reference	
Falls from height	0.88 (0.32-2.16, *P* = 0.779)	
Other	4.25 (0.19-46.84, *P* = 0.248)	
ASIA scale at admission		
E	Reference	Reference
A-D	341.46 (64.37-6354.54, *P* < 0.001)	311.42 (57.84-5835.85, *P* < 0.001)
Admission to ICU^c^	6.66 (2.23-18.96, *P* < 0.001)	4.14 (0.72-37.47, *P* = 0.147)
Site of injury		
Cervical^c^	3.55 (1.47-8.67, *P* = 0.005)	#
Thoracic^c^	1.70 (0.67-4.10, *P* = 0.244)	
Lumbar^c^	0.30 (0.11-0.74, *P* = 0.011)	#
Multiple associated injuries		
Head injury^c^	1.35 (0.20-5.32, *P* = 0.705)	
Chest injury^c^	5.16 (1.48-16.19, *P* = 0.006)	#
Orthopedic injuries^c^	1.63 (0.56-4.20, *P* = 0.336)	
Visceral injury^c^	NA	
Maxillofacial injuries^c^	NA	

## Discussion

TVSIs are a well-recognized cause of long-term morbidity and mortality. In our study, the patients with a TVSI had a median age of 35 years and a male-to-female ratio of 4:1, both of which are congruent with the literature [6-9]. The male-to-female ratio is close to the average global ratio of 3.4:1 [4]. However, it is much lower than other studies conducted in Saudi Arabia, which have reported a ratio as high as 7.5:1 [21]. This can be explained by the fact that women have only been allowed to drive since June 2018, so men had more exposure to RTAs before that time [22]. Similar to the findings by Alawad et al., Saudis constituted the majority of our sample, and an RTA was more likely to be the mechanism of injury compared with that of non-Saudis [6]. Falls from height were observed more among non-Saudis [6]. The lumbar site was the most affected spinal area, followed by the thoracic and then the cervical sites [6]. This differs from studies by AlEissa et al. and Bakhsh et al., as well as the global study by Kumar et al., which found the cervical region to be the most affected area [4,9,23]. About 44% of our sample required surgery, which is very similar to the global study by Kumar et al. and the Saudi study by Alkhathlan et al. [4,7].

In this study, the most common mechanism of injury was an RTA, followed by a fall from height. Saudi Arabia has the highest reported proportional rate of TVSI caused by an RTA worldwide, followed closely by Qatar [24]. Common causes of RTAs include the use of mobile phones while driving, driving at high speeds, and drifting [21,25]. Thus, stricter enforcement of driving rules is needed, along with widespread campaigns to educate young people about the risks and consequences of reckless driving. Saudi Arabia has witnessed a notable improvement in the enforcement of traffic rules recently. We believe this is going to decrease RTAs in the future. TVSIs due to falls from height accounted for 32.1% of the cases in our study. Similar results were observed in other studies: a study by Mehdar et al. in Najran, Saudi Arabia, showed that falls were responsible for 23% of TVSI cases [26].

The ASIA score is one of the most important grading systems commonly used to evaluate a TVSI [19,20]. The majority of patients (83%) in our study had an ASIA score of E upon admission, while the remaining 17% had scores of A through D. At discharge, 90% of patients had an ASIA score of E. An ASIA score of A through D was the only determinant associated with receiving an ASIA score of A through D at discharge. Although most patients did not have a change in their ASIA scores from admission to discharge, it should be noted that an improvement in the ASIA score should not be used as a predictor of the prognostic functional capacity of a person with a TVSI because it is poorly associated with future ability to ambulate after a TVSI [27]. We classified functional disability as either paraplegia or tetraplegia. In our study, 3.8% of the patients had paraplegia, while 2.5% had tetraplegia, which is consistent with the literature [26].

LOS is a commonly adopted outcome measure for effective TVSI care [14-16]. The National Spinal Cord Injury Statistical Center (NSCISC) in the United States showed a remarkable decline in LOS from 24 days in the 1970s to 11 days in 2016 [28]. In our study, the median LOS in acute care was 10.0 days. Of note, some studies have investigated several determinants of LOS; prolonged LOS was found more among older patients; those with vertebral injury, pneumonia, or pressure ulcers; and those who had had late surgical management [14,17,18]. In our study, we found no significant effect of age on LOS. Furthermore, our participants had a very low rate of secondary complications, so those were not included in the model. We found that being non-Saudi, having an ASIA score of A through D at admission, and having related orthopedic injuries were associated with a prolonged LOS.

This study had a retrospective design and was conducted in a single center, which might affect the generalizability of the results. However, this effect should not be very pronounced as the hospital where the study was conducted (BCH) has one of the largest neurosurgical departments in the region, and many neurosurgical patients from regional hospitals are referred there. We were met with other obstacles, including missing data related to the length of the rehabilitation period, length of surgical management, and other functional outcome measures, such as the Walking Index for Spinal Cord Injury (WISCI II) [29] and the functional independence measure (FIM) [30]. Adopting a centralized nationwide database for TVSIs, like that of the NSCISC in the United States or the Norwegian Spinal Cord Injury Registry (NorSCIR), would help standardize the reporting and collection of TVSI data. Furthermore, it would facilitate more population-based studies to understand the root causes of the problem in Saudi Arabia.

## Conclusions

This study demonstrated the characteristics of and outcomes for patients with a TVSI in the Al-Qassim region in Saudi Arabia. We identified the determinants of an ASIA score of A through D and the LOS in hospital acute care. As most TVSIs in Saudi Arabia are caused by RTAs, traffic rules should be more strictly enforced to better control the situation. Furthermore, we urge the development of a centralized nationwide database in Saudi Arabia to better understand TVSI cases in the kingdom.

## References

[1] Rubiano AM, Carney N, Chesnut R, Puyana JC (2015). Global neurotrauma research challenges and opportunities. Nature.

[2] Reilly P (2007). The impact of neurotrauma on society: an international perspective. Prog Brain Res.

[3] Zhang S, Wadhwa R, Haydel J, Toms J, Johnson K, Guthikonda B (2013). Spine and spinal cord trauma: diagnosis and management. Neurol Clin.

[4] Kumar R, Lim J, Mekary RA (2018). Traumatic spinal injury: global epidemiology and worldwide volume. World Neurosurg.

[5] (2021). World Health Organization: Global Status Report on Road Safety 2018: Summary. https://apps.who.int/iris/handle/10665/277370.

[6] Alawad MO, Alenezi N, Alrashedan BS, Alsabieh M, Alnasser A, Abdulkader RS, Surur S (2020). Traumatic spinal injuries in Saudi Arabia: a retrospective single-centre medical record review. BMJ Open.

[7] Alkhathlan KM, Alzahrani MG, Aldosari KH, Alsheddi MI, Alqeair AA (2019). Traumatic spinal injuries in the Kingdom of Saudi Arabia: a study of associated injuries, management and mortality. Pan Afr Med J.

[8] Aldosari KH, Aldhfyan YM, Karrar MH, Aldossary AM, Deailj AA, Al-Ameer KH, Alsubaie ML (2019). Severity and neurosurgical management of patients with traumatic spinal fractures in Saudi Arabia: a cross sectional study. Pan Afr Med J.

[9] Bakhsh A, Aljuzair AH, Eldawoody H (2020). An epidemiological overview of spinal trauma in the Kingdom of Saudi Arabia. Spine Surg Relat Res.

[10] Al-Habib A, Alaqeel A, Marwa I (2014). Causes and patterns of spine trauma in children and adolescents in Saudi Arabia: implications for injury prevention. Ann Saudi Med.

[11] Al-Jadid MS (2013). A retrospective study on traumatic spinal cord injury in an inpatient rehabilitation unit in central Saudi Arabia. Saudi Med J.

[12] Al-Jadid M, Robert AA (2010). An analysis of the length of stay in traumatic and non-traumatic spinal cord injured patients. A rehabilitation unit experience in Saudi Arabia. Saudi Med J.

[13] Barrimah I, Midhet F, Sharaf F (2012). Epidemiology of road traffic injuries in Qassim region, Saudi Arabia: consistency of police and health data. Int J Health Sci (Qassim).

[14] Dvorak MF, Noonan VK, Fallah N (2015). The influence of time from injury to surgery on motor recovery and length of hospital stay in acute traumatic spinal cord injury: an observational Canadian cohort study. J Neurotrauma.

[15] Tooth L, McKenna K, Geraghty T (2003). Rehabilitation outcomes in traumatic spinal cord injury in Australia: functional status, length of stay and discharge setting. Spinal Cord.

[16] Burns AS, Santos A, Cheng CL (2017). Understanding length of stay after spinal cord injury: insights and limitations from the access to care and timing project. J Neurotrauma.

[17] Conradsson D, Phillips J, Nizeyimana E, Hilliar C, Joseph C (2019). Risk indicators of length of acute hospital stay after traumatic spinal cord injury in South Africa: a prospective, population-based study. Spinal Cord.

[18] Furlan JC, Noonan V, Cadotte DW, Fehlings MG (2011). Timing of decompressive surgery of spinal cord after traumatic spinal cord injury: an evidence-based examination of pre-clinical and clinical studies. J Neurotrauma.

[19] Roberts TT, Leonard GR, Cepela DJ (2017). Classifications in brief: American Spinal Injury Association (ASIA) impairment scale. Clin Orthop Relat Res.

[20] van Middendorp JJ, Goss B, Urquhart S, Atresh S, Williams RP, Schuetz M (2011). Diagnosis and prognosis of traumatic spinal cord injury. Global Spine J.

[21] Alshahri SS, Cripps RA, Lee BB, Al-Jadid MS (2012). Traumatic spinal cord injury in Saudi Arabia: an epidemiological estimate from Riyadh. Spinal Cord.

[22] (2021). Saudi Arabia Agrees to Let Women Drive. https://www.nytimes.com/2017/09/26/world/middleeast/saudi-arabia-women-drive.html?utm_medium=email&utm_source=transaction.

[23] AlEissa S, AlAssiri SS, AlJehani RM (2019). Neurological disability among adults following traumatic spinal fractures in Saudi Arabia: a retrospective single-center medical record review. Ann Saudi Med.

[24] Lee BB, Cripps RA, Fitzharris M, Wing PC (2014). The global map for traumatic spinal cord injury epidemiology: update 2011, global incidence rate. Spinal Cord.

[25] Ansari S, Akhdar F, Mandoorah M, Moutaery K (2000). Causes and effects of road traffic accidents in Saudi Arabia. Public Health.

[26] Mehdar KM, Mahjri AA, Al Rashah AA, Alyazidi A (2019). Epidemiology of spinal cord injuries and their outcomes: a retrospective study at the King Khalid Hospital. Cureus.

[27] van Middendorp JJ, Hosman AJ, Pouw MH, Van de Meent H (2009). ASIA impairment scale conversion in traumatic SCI: is it related with the ability to walk? A descriptive comparison with functional ambulation outcome measures in 273 patients. Spinal Cord.

[28] (2021). National Spinal Cord Injury Statistical Center: Facts and Figures at a Glance. https://www.nscisc.uab.edu/Public/Facts%20and%20Figures%202020.pdf?utm_medium=email&utm_source=transaction.

[29] Dittuno PL, Ditunno JF, Jr Jr (2001). Walking index for spinal cord injury (WISCI II): scale revision. Spinal Cord.

[30] Keith RA, Granger CV, Hamilton BB, Sherwin FS (1987). The functional independence measure: a new tool for rehabilitation. Adv Clin Rehabil.

